# Continuous wave high-power laser propagation in water is affected by strong thermal lensing and thermal blooming already at short distances

**DOI:** 10.1038/s41598-021-02112-6

**Published:** 2021-11-19

**Authors:** Stefan Reich, Sebastian Schäffer, Martin Lueck, Matthias Wickert, Jens Osterholz

**Affiliations:** Fraunhofer Institute for High-Speed Dynamics, Ernst-Mach-Institut, EMI, Ernst-Zermelo Straße 4, 79104 Freiburg, Germany

**Keywords:** Applied physics, Fluid dynamics, Optical physics, Laser material processing, Imaging and sensing

## Abstract

When laser beams propagate through media with non-vanishing absorption, the media is heated resulting in a change of the refractive index, which can lead to thermal lensing and thermal blooming. However, experimental details about both phenomena for propagations in water are lacking, especially for high-power lasers in the kilowatt range. We show that significant thermal lensing occurs only for high input powers before the onset of convective flow, while for low input powers, no strong thermal lens arises. After the onset of water flow, thermal blooming occurs at low input powers comparable to that known for propagations over kilometres in the air. However, for high input powers a thermal blooming on a qualitatively higher level is shown. By wavefront sensing, the change of refractive index distribution in water is investigated. This clearly shows the fast development of a strong thermal lens for high input powers and the onset of convection. Furthermore, a qualitatively good agreement of the accompanying simulations is observed. It is found that the absorption coefficient is linear with a value of $\mu ={13.7}\,{\mathrm{m}^{-1}}$ at least up to 7.5 kW, i.e. 8 $\mathrm{kW/cm}^2$. However, the directed transmission into an aperture is only constant before any thermal lensing of blooming occurs.

## Introduction

Thermal lensing is known to occur for laser beams that propagate in transparent media without vanishing absorption^1–4^. Due to the deposited energy, the media temperature increases resulting in a locally changed refractive index. Since the refractive index in water decreases with increasing temperature^5^, a diverging lens is formed. As long as there is no movement of the medium, the assumption of a symmetrical beam profile results in a constant symmetrical distribution of the refractive index and thus also of the thermal lens^6^.

A medium movement occurs in fluid or gaseous media and is either driven externally by e.g. wind^7^ or internally by the onset of a convective flow^8^. Then, the thermal lens is distorted, resulting in non-symmetric transmitted beam shapes. This is called thermal blooming and is mainly investigated for beam propagation in air^9–11^. Many investigations have been carried out using simulation in air^8, 12, 13^ and have mainly been driven by the goal to overcome the beam distortion by compensation with adaptive optics^14, 15^. For laser beam propagation in liquids, only minor investigations do exist in experiment^7, 16^ and simulation^4, 17^. Investigations for water and high-power lasers in the kilowatt range are lacking completely. Compared to propagation in air, the absorption coefficient in water is much higher^8, 18^ resulting in an increased thermal blooming effect. Therefore, the required propagation distance to obtain a similar beam distortion is strongly decreased.

A direct investigation of the propagation medium in the path of high-power lasers is not possible, since all devices could interact with the laser and be destroyed by the high thermal load. Therefore, indirect techniques or suitable simulations are required. The refractive index of an optical element can be measured indirectly with a wavefront sensor^19^. However, most techniques require multiple partial measurements to determine a wavefront distortion. This is inappropriate for fast processes. Techniques based on the Hartmann-mask principle^20^ can be single-exposure measurement methods^21^. In this case, a reference image must be acquired before starting the experiment, and then only one image per acquisition is needed to determine the wavefront distortion. From the local shift of the Hartmann-mask structures, the wavefront distortion due to refractive index inhomogeneities can be calculated. For investigations with a large field of view, wavefront sensors that image directly onto a focal plane array are not convincing because large detectors are required^3, 22, 23^.

With this work, we aim to fill a gap in our knowledge about the physical basis of the occurrence of thermal lensing and thermal blooming in water, especially at high laser intensities. Possible applications arise in the field of defusing of explosives by deflagration under water^24^. In this context, a certain working distance must be maintained for safe laser processing. Therefore, knowledge about opportunities and restrictions of laser processing with high powers through water columns is necessary. By imaging the intensity distribution of the laser spot after propagation through a water layer, we show the occurrence of thermal blooming for lower input powers. For high laser powers, the establishment of the thermal lens is faster than the onset of water movement. Thus, initially a strong thermal lens is created followed by a strong irregular beam distortion. While for low laser powers the transmitted intensity distribution is similar to the well known thermal blooming^7, 8^, the distortions for high laser powers are much stronger showing a new level of thermal blooming above the known moderate levels.

The change of the refractive index distribution in water is determined by means of Hartmann-mask wavefront sensing. Due to the perpendicular orientation of the wavefront sensor, both the thermal lens evolution and the convective flow are detected. The accompanying simulations show comparable results, confirming their validity. This provides a more complete picture of the changing parameters such as refractive index distribution and temperature in the water.

In addition to the beam distortion, the effect of the absorption of the laser energy in the water is analysed. The transmitted output power and intensity distribution ultimately is of interest to understand the effects of high-power lasers to target materials^25, 26^ behind a water layer. The absorption coefficient for the used wavelength of 1070 nm is reported to be between 12 ${\mathrm{m}^{-1}}$ and 14.8 ${\mathrm{m}^{-1}}$ in literature^18, 27–29^. The absorption only decreases slightly when the water is heated^28, 30^. However, these absorption coefficient values are normally determined at low intensities. Until now, it has not yet been investigated whether these values are still valid at high intensities which are typical for high-power lasers. Here we describe the absorption of cw laser light in the kilowatt range with intensities of up to 8 $\mathrm{kW/cm}^2$.

## Experimental setup

**Figure 1:**
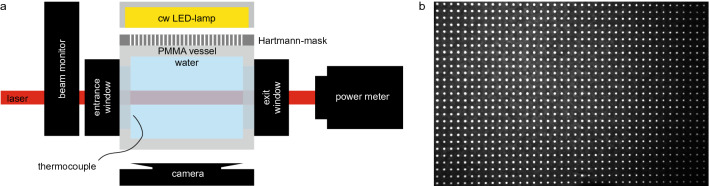
(**a**) Experimental setup for measuring the transmission of laser light in water and the refractive index changes. The laser spot shape was measured in front of the water vessel via a beam monitor, while the transmitted power was determined with a power meter. For imaging the transmitted intensity distribution, the power meter was replaced by an aluminium plate as scattering screen (tilted by 45$^{\circ }$ to the laser direction) and was imaged with a camera. The temperature increase in the water was detected with a thermocouple. The wavefront distortion was measured with a custom-made Hartmann-mask detector consisting of a LED-lamp, a rectangular spot pattern screen (**b**) and a camera imaging the spot pattern.

In Fig. 1 the experimental setup is illustrated. The main part consisted of a custom-made water vessel made of Poly(methyl methacrylate) (PMMA) with a water penetration depth of 10.1 cm. Ordinary tap water was used, which does not reflect the purity required for metrology, but rather reflects everyday usage. The two windows were uncoated fused silica glass windows of 50.8 mm diameter and 12 mm thickness (WG42012, Thorlabs GmbH, Germany). They have each a transmission of 93.5% at a wavelength of 1070 nm resulting in a decrease of transmitted power of 13%. The transmitted laser power was measured with a “PowerMax-Pro 3 kW Sensor” (Coherent Inc., USA). The entrance aperture of the power meter has a diameter of 30 mm. The sensor element is located 57 mm behind the entrance, which leads to a distance of around 80 mm between the end of the water phase and the detector.

The laser used was an ytterbium fiber-laser (YLS-10000, IPG Photonics Corporation, Germany) with a wavelength of 1070 nm, a line width of 5nm and a maximum average power of 10 kW (1% power stability). It was operated in continuous wave mode. It consists of 18 single-mode laser modules, which are spliced into a feeding fiber with a 100 ${\upmu }{\mathrm{m}}$ core diameter. To protect the feeding fiber from mechanical damage, this fiber is coupled into a multimode processing fiber with a core diameter of 200 ${\upmu }\mathrm{m}$. The output laser beam exhibits a beam quality factor of $M^2=18$. The switching on/off time of the laser is around 40 ${\upmu }\mathrm{s}$. The spot size at the sample position was changed with a custom made zoom optics (Reis Lasertec GmbH, Germany). It allows focusing the laser between 5m and 50m by moving one lens in the optic. Before entering the water vessel (4.25 m from the zoom optics), the laser spot shape was measured with a beam monitor BM60 (PRIMES GmbH, Germany). Three different values of the beam diameter ($D4\sigma$) of 10.2, 17.8 and 25.1 mm were used for the experiments. The focus distances were roughly 4.8, 5.4 and 6.0 m, respectively. The beam had an almost circular Gaussian shape.

The temperature of the water was measured with a type K thermocouple (SCASS-020E-6, Omega Engineering GmbH) with a signal conditioning unit TSA-TC2-K-T12-1k/30-BW-V2/A3 (Imtron Messtechnik GmbH, Germany). The tip of the thermocouple was placed at the beginning of the vessel next to the laser spot to not irradiate the thermocouple. The signals of the laser control output, the power meter and the thermocouple were recorded with a transient recorder TransCom-CompactE-XL (MF Instruments GmbH, Germany) with a 10 kHz frame rate.

The local distribution of the transmitted laser beam intensity was measured in a separate experiment by imaging the laser light scattered from an aluminium plate with a “PCO edge 5.5” (PCO AG, Germany) with a “Nikkor AF 24-85mm” lens (Nikon GmbH, Germany), operated in gating mode with an internal frame rate of 100 Hz and an exposure time of ${9}\,{\upmu }\mathrm{s}$. The plate was located 13 cm behind the exit window and was tilted by 45$^{\circ }$ to the beam direction.

Lasing time was modulated in two ways: First, in order to determine the transmitted laser power, pulse trains of 5 pulses of 0.1 s duration and 10 Hz repetition rate were used. The primary laser power was changed from 1 to 10 kW. To account for the reduced transmission inside the zoom optics and the water vessel windows, the transmission without water was determined with the same pulse trains for primary laser powers of 1, 2 and 3 kW as well. For analysis purposes, either the average transmission values of the complete laser-on times or only a single point in time at the beginning of the first pulse were used. Second, in order to further investigate the transmission, temperature increase, refractive index changes and particularly the temporal intensity distribution of the transmitted laser beam, single pulses of 5 s duration with primary laser powers of 1, 2, 5, 7 and 10 kW were used. For the results shown, the transmission corrected input power at the beginning and the output power at the end of the water phase are always stated. Due to the partly strong heating of the water, it was replaced after each laser pulse series and 5 s long single pulse.

To investigate the change of the refractive index distribution, a wavefront measurement with a probe beam transversal to the high-power laser was performed. With the multi-contrast imaging technique based on a Hartmann-mask^20^, three contrast types can be obtained in single exposure acquisition: absorption, scattering and differential phase. The latter is of importance to investigate fast changing systems. The broad beam of a continuous wave LED light (Constellation 120E, IDT Inc., USA) is split into a bunch of beamlets when passing the Hartmann-mask. The latter was realized with a printed black paper with white dots on it (see an image in Fig. 1b). The 2D spot pattern was a Cartesian grid with a spacing of 2 mm and spot diameters of 0.5 mm. This pattern was measured with a “Phantom v2640” (Vision Research Inc., USA) high-speed camera with a frame rate of 1 kHz and an exposure time of ${3}\,{\upmu }$s and a “Nikkor AF 24-85mm” lens (Nikon GmbH, Germany). A change in the distribution of the refractive index in the water phase results in shifted positions of the bright spots on the camera. The shift of the spots represents the differential phase of the optical element, in detail the integrated refraction power along the beamlet path. The analysis is based on a Fourier analysis^31, 32^. A detailed description of the self written code can be found elsewhere^21, 33^. In brief, the images were Fourier transformed, the zero and first harmonic orders were cropped and transformed back. For the investigations in this paper, the differential phase contrast is of interest, which was obtained by the change in the complex angle of the first harmonic order between a reference image (before laser turned on) and the measurement images.

## Simulation of laser absorption and Hartmann-mask imaging

In the experiment, only minor information about the temperature distribution and hence distribution of the refractive index as well as the convective flow during the laser irradiation can be obtained. For example, the temperature can only be measured outside the laser beam path. Furthermore, by the phase contrast imaging, only the effect of a cumulative change in the refractive index along the beamlet path can be monitored. To obtain more information about the laser induced changes of temperature distribution and flow velocity in the water, the experiments were simulated with COMSOL Multiphysics 5.6 (Build: 280) (Comsol Multiphysics GmbH, Germany). The simulation consisted of two parts: first, the absorption of the laser in the water with consequent heating up and water flow. In the second step, ray tracing was applied to determine beamlet deflections due to the local inhomogeneous refractive index distribution.

The modules COMSOL Multiphysics, CFD, Heat Transfer and Ray Optics with the physic modules “radiative beam in absorbing media”, “heat transfer in fluids”, “laminar flow” and “geometrical optics” were used. Furthermore, the multiphysics modules “heat transfer with radiative beam in absorbing media” for coupling the “heat transfer in fluids” with “radiative beam in absorbing media” and “nonisothermal flow” to couple the “laminar flow” with the “heat transfer in fluids” were used. The laminar flow was done with a “weakly compressible flow”, inclusion of gravity and no turbulences. The wall condition was “no slip”. The choice of the laminar flow solver was motivated by a first set of simulations, where laminar and turbulent flow models were compared. It was found that there was no significant difference between the different flow models. Therefore, the laminar flow model was used for further data analysis, since it requires less computational time. Simulating the turbulence observed in some of the experimental data would require a more complex analysis and is beyond the scope of this paper.

The water geometry was similar to the one in the experiments with a rectangular box of the size ${6\times 7\times 10}$ cm^3^ for the width, height and depth from the perspective of the laser. The meshing of the water was done with the predefined size “extremely fine”. The walls of the water were set as thermally isolated. The distance from the end of the water to the ray detecting wall of the ray tracing was 15 cm. This is a similar distance compared to the imaging of the transmitted intensity distribution. To simulate the laser beam shape after transmission, a ray tracing parallel to the laser beam over the complete water vessel area was performed with a rectangular ray grid of spacing 0.5 mm. The intensity distribution was propagated from the starting points of the rays to the endpoints to calculate the intensity distribution after water transmission.

The axis of the incident laser beam was oriented horizontally and was placed through the center of the simulation volume at a height of 3 cm. The beam shape used was a Gaussian distribution with standard deviation $\sigma ={4.45}\,\mathrm{mm}$. This corresponds to the beam diameter $D4\sigma = {17.8}\,\mathrm{mm}$ used in the experiments. However, the simulation represents a perfectly collimated beam and not the converging beam of the experiments.

As material parameters, the build-in parameters for “H2O (water) [liquid]” were used except for the absorption coefficient, the refractive index and the ratio of specific heats, which was set to 1. The absorption coefficient of water was $\mu = {13.7}\,{\mathrm{m}^{-1}}$, which corresponds to the value determined in the experiments (see below). The temperature-dependent index of refraction *n*(*T*) was calculated by: $n(T) = 1.06 + 2.55\cdot 10^{-3} \cdot T -7.27\cdot 10^{-6} \cdot T^2 + 6.29\cdot 10^{-9} \cdot T^3$, where the temperature *T* was in units of Kelvin. The parameters were determined by fitting the tabulated values for a wavelength of 404nm between 0$^{\circ }$C and 90$^{\circ }$C at 1bar pressure from Schiebener et al.^5^. Although this formula does not represent the absolute value of the laser at a wavelength of 1070 nm, it is noted that here only the change of the refractive index and not the absolute value is of interest. Therefore, the differences for the absolute values compared to the laser wavelength of 1070 nm is irrelevant. The change of the refractive index with temperature is almost independent of the wavelength in the region of interest of this paper. The starting value for the temperature was 293.15 K.

To simulate the Hartmann-mask imaging of the experiments, a ray tracing with a rectangular grid of initially parallel rays was used. Therefore, the refractive index distribution in the water was calculated from the temperature map as stated above. A grid of rays was released at a spacing of 2 mm, the Hartmann-mask spot distance, over the complete area of the water vessel. The starting point of the rays was the one vessel side with a perpendicular direction into the water. After penetration through the water, the rays were stopped at a detector wall placed 15 cm behind the water. Since the beam deflection was calculated, the absolute value of the free propagation between the water and the detector wall is not relevant.

The simulation was done in two consecutive studies. First, the absorption of the laser beam in the water phase with the included thermal liquid changes was studied. The time was changed from 0 to 5 s in steps of 0.1 s. The study time steps were 0.005 s. In the second step, the ray tracing was performed on basis of the results of each time step of the first study. The time steps for the ray tracing were 0.002 ns. The analysed time steps were 0 ns and 2 ns representing the start at the Hartmann-mask and the end after detection in the camera. However, for the second time step, all rays were already frozen at the walls acting as detectors.

## Results and discussion

### Transmission calibration of the setup

Both the zoom optics and the windows of the water vessel have a finite transmission. To take this into account, the setup transmission was determined by detecting the transmitted power of only the zoom optics and the zoom optics together with the empty water vessel. The zoom optics has a transmission of 80% and each of the two vessel windows one of 93.5%. The primary laser power and the measured power with the power meter are thus corrected for the transmission of the zoom optics and the vessel windows. In the following, the laser power at the beginning and at the end of the water phase are always given as “input power” and “output power”. The input power thereby represents 75% of the primary laser power. The output power before the exit window is 106.5% of the power measured by the power meter.

**Figure 2:**
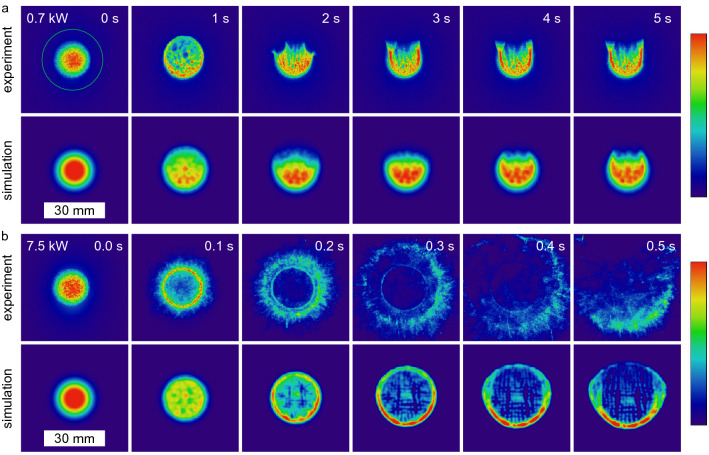
Intensity distribution (colorbar on the right, logarithmic scale, arb. u.) of a laser spot ($D4\sigma ={17.8}\,\mathrm {mm}$, input power 0.7 kW (**a**) and 7.5 kW (**b**), continuous wave) after passing 10.1 cm water determined by experiments and simulations. For the lower input power, first a size increase and then an opening towards the top is observed. The green circle indicates the aperture size of the used power meter. For the high input power, a much faster change over a ring pattern to a fast changing and irregular large pattern is observed. Phenomenologically, there is a good agreement between the experimental and simulated results, considering that in the simulation the change of the refractive index was not taken into account for the energy deposition (see text for explanation).

One further aspect of the calibration of the setup was to exclude the fact that the glass windows alone cause a significant thermal lensing effect. Therefore, the intensity distribution behind the empty vessel with only the glass windows was recorded analogously to the investigations with the water (see Fig. 2). No change in the intensity distribution was observed.

### Transmitted intensity distribution—thermal lensing and thermal blooming

For laser beams transmitted through fluid media with non-vanishing absorption, it is known that thermal blooming occurs^8, 34^. It originates from refractive index variations due to medium heating by the laser in conjunction with a movement of this heated medium. This movement results either from external conditions like wind in air or from internal heat-induced convection. The latter is taking place here, as no external flow exists. The water efficiently absorbs the laser light, resulting in rapid heating of the water and consequent convective flow.

In Fig. 2 the temporal changes of intensity distribution for two input powers are shown. The laser (continuous wave for 5 s) spot size was $D4\sigma ={17.8}\,\mathrm {mm}$. The magnitude of change thereby scales with the input power. The initial intensity distribution is Gaussian. For 0.7 kW input power (Fig. 2a), after 0.4 s the spot size increases and shows local intensity maxima distributed over the complete area of the spot. It changes from Gaussian shape to a locally inhomogeneous flat-top shape. After 1 s it starts to open at the top, hence the center of the intensity distribution moves downwards. After around 2 s a U-like shape starts to form which changes only sightly thereafter. This behaviour is well known as thermal blooming, mostly investigated in air^8, 12, 35^. As air has got a much smaller absorption coefficient compared to water, the propagation distances are much larger in these cases. However, the presented low input power intensity distributions after the short propagation distance in water look very similar.

A much faster and different change of the intensity distribution is observed for the high input power, as can be seen in Fig. 2b. The Gaussian shape deforms to a flat-top within 30 ms, coming to a ring shape after 100 ms. Subsequently, the main part of the laser power is located outside the region of the original laser spot. Due to the high input power, the water is heated very quickly. The laser spot initially has a Gaussian shape, which leads to a radially symmetric gradient in the temperature field and consequently also in the distribution of the refractive index. The refractive index decreases with increasing temperature^5^ leading to a diverging lens. In the high-power range, this process is so fast and strong that within 200 ms a thermal lens is formed that is so strong that it displaces almost all of the radiation from the primary spot region. A similar behaviour was previously found for laser propagation in ethanol^4^. No significant water flow has formed during this time, resulting in the nearly round intensity distribution. However, due to the high temperature gradients a significant water flow forms afterwards. The simulations specify a mean velocity of 2.5 cm/s within the initial beam diameter and a velocity of 4 cm/s in the initial center of the spot as maximum value (data not shown). This constant flow forms within 1 s. Then, a more or less stationary distribution of the temperature and refractive index arises within the laser path. From below, cold water flows into the region of the laser path, heats up and continues to flow upward. Therefore, the shape of the transmitted intensity varies only slightly after about 0.5 s. A laying half moon is observed at the bottom with fast changing but low intensity parts at the top.

The simulations of the experiment show a qualitatively good agreement. The intensity distribution after water penetration changes slightly for low input powers and strongly for high input powers (see Fig. 2). The simulations are limited by the fact that no completely coupled simulation is possible, since for the energy deposition simulated by the “radiative beam in absorbing media” module, the changing refractive index distribution cannot be taken into account. Nevertheless, the beam size enlargement and subsequent deformation to a U-like shape is very similar for the low input power. Moreover, for the high input power, the transformation of the intensity distribution from Gaussian over a ring to a broad U-shaped form can be properly simulated. A slight temporal difference between the experiments and the simulations can be observed here. The highly resolved intensity distributions in the experiment are not resolved in the simulations due to the limited resolution.

### Determination of the refractive index changes by Hartmann-mask wavefront measurement

**Figure 3:**
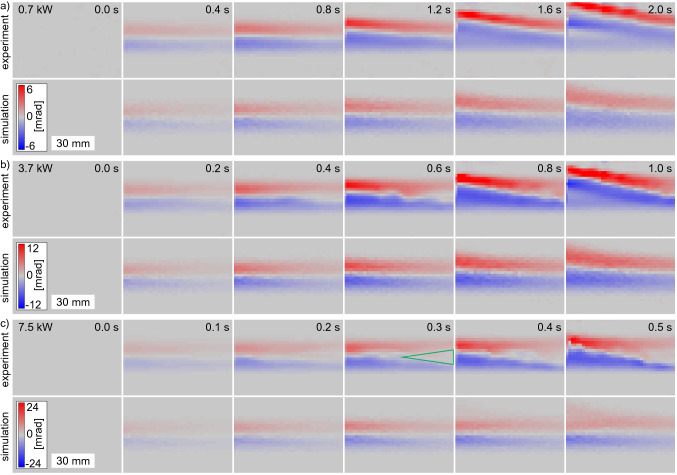
Differential phase, hence beamlet deflection (positive values represent deflection upwards and vice versa) of horizontal probe beam perpendicular to the high-power laser. Deflection arises due to refractive index changes because of a temperature increase during laser propagation through water. The laser (0.7, 3.7 and 7.5 kW, $D4\sigma ={17.8}\,\mathrm {mm}$, continuous wave) is coming from the left. For all input powers an increasing beamlet shift with time and towards the entrance of the water vessel is observed. For 3.7 kW and 7.5 kW, a decreased shift is obtained in the center of the laser path in the back part of the water (green triangle as guide to the eye) due to a significantly decreased energy deposition there because of the thermally induced divergent lens. For 0.7 kW only a homogeneous spot shift increase is observed. After a certain time a convective flow sets in shown here by the upward movement of the area of spot shift. The simulations show a qualitative good agreement with the experimental results.

With the differential phase contrast of the multi-contrast imaging by a Hartmann-mask, the change of the refractive index distribution can be visualised. The beamlets of the Hartmann mask get deflected by refractive index inhomogeneities as they pass through the water. Note that only continuous changes and no step function of the refractive index can be measured with this technique, which is valid for these experiments. Furthermore, it has to be mentioned that the beamlet deflection is the integrated refraction power over the complete penetration path of the beamlets. In Fig. 3 the differential phase maps for the three input powers 0.7, 3.7 and 7.5 kW (continuous wave for 5 s) are shown. Note the different time steps and absolute scaling. At the beginning a homogeneous distribution is observed with stronger beamlet deflections at the entrance side (left) compared to the rear side for all input powers. The beamlets get deflected upwards in the top half and downwards in the bottom half of the laser path. This indicates a diverging lens, which is in agreement with the expectations. As already discussed above for the transmitted intensity shape, the heating of the water results in a decrease in refractive index which is stronger in the center of the laser path and close to the entrance window of the water vessel. This represents a diverging lens in laser propagation direction as well as perpendicular to it. This diverging lens gets stronger with time. After a certain time, which is shorter for higher input powers, this heat affected water zone with decreased refractive index starts to move upwards. These times coincide well with the times when the transmitted intensity distributions start to leave radial symmetry. This change of the refractive index directly explains the emergence of the thermal lensing and blooming shown in Fig. 2. Before the onset of the water flow, the locally stable but in strength increasing diverging lens is the reason for the enlargement and the deformation of the intensity profile from Gaussian to flat-top. At the time when the water flow sets in, the transmitted intensity profiles also opens at the top resulting in thermal blooming.

For the high input powers, a much stronger thermal lensing is observed in Fig. 2b. And also the differential phase map clearly shows the fast and strong development of a thermal lens. While for the low input power, the differential phase map shows a homogeneous diverging lens over the complete laser path, this is not the case for the high input powers. There, a region of almost no change of the refractive index is present in the center of the rear part of the laser propagation path (see illustrated triangle at 0.3 s for 7.5 kW input power in Fig. 3). While energy is deposited at the front over the complete laser spot area for longer times, this happens at the rear part only at the beginning before the laser beam gets deflected outside of the original laser path due to the strong thermal lens.

As in the case of the intensity distribution, the simulations of the Hartmann-mask measurements also show good agreement with the experimental results. The comparison of the various differential phase maps in Fig. 3 shows a similar development of the diverging lens in strength and time. Minor differences are observed as the experimental data show a higher amount of turbulent flow especially after the onset of the water flow. The absence of the minor heated central part of the laser path at the rear side of the water clearly shows the current limitations of the simulation. The local energy deposition does not take into account the changed distribution of the refractive index and thus a changed energy deposition due to the resulting thermal lens. Nevertheless, the good agreement shows the validity of the simulations.

### Absorption of laser power in water

#### Short laser pulses of 0.1 s

**Figure 4:**
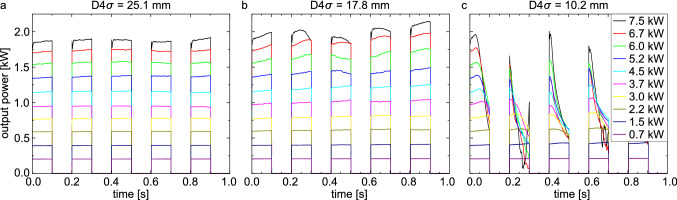
Output power as power in the bucket (aperture diameter 30 mm) after a water column of 10.1 cm with different laser spot sizes and input powers. For large spot sizes and low input powers, the power in the bucket is constant with time while it varies strongly for small spot sizes and high input powers. The latter arise from thermal lensing and thermal blooming.

As discussed before, the intensity distribution of a high-power laser beam radiating through a water column changes with time. The water absorbs light of a wavelength of 1070 nm comparatively strongly, which leads to thermal lensing and thermal blooming due to water heating. For a technical usage of high-power lasers for under water processing, the deposited energy at a final target is of central interest. In Fig. 4 the output power measured within the aperture of the power meter, hence the power in the bucket, is shown. Short pulse trains with different input powers and spot diameters were used. For all three spot diameters, the power in the bucket scales with the input power. However, both the laser power and the spot diameter do influence the temporal behaviour of the power in the bucket.

For the largest spot diameter, plotted in Fig. 4a, the power in the bucket starts to change with time only for the highest input powers used. Above an input power of 3.7 kW, the power in the bucket starts to change slightly with time. Even for the highest input power of 7.5 kW a variation of less than 2% is observed within the pulse trains. For the smallest spot diameter in Fig. 4b, the input power at which a significant change in the power in the bucket occurs drops below 1.5 kW. And especially for the higher input powers and the smallest spot diameter, the power in the bucket strongly decreases even within the short duration of 0.1 s of a single pulse.

This variation of the power in the bucket must be attributed mainly to the spatial distortion of the laser beam in the water. As discussed above, thermal lensing and thermal blooming do occur very fast. Only a small uncertainty of less than 2% was observed in the transmission calibration of the setup. For the small spot size, the intensity limit of the used power meter is already reached, which may have caused this small uncertainty. Moreover, a significant change in the power in the bucket at the beginning of each pulse within a pulse train is only observed in the experiments with water, but not in the transmission calibration.

As already shown, the intensity distribution of the transmitted laser beam changes very fast for high input powers. In Fig. 2 the spot diameter used was $D4s={17.8}\,\mathrm {mm}$ which corresponds to the medium diameter of the following investigations of the absorption. For the highest input powers, a significant diverging lens forms already within 0.1 s. For the smallest spot diameter, the input intensity is higher by a factor of 3. Therefore, it is not surprising that already within the first pulse of 0.1 s the detected power in the bucket strongly decreases. The thermal lens arises within a period of less than 100 ms directing the transmitted power outside of the aperture of the detector.

**Figure 5:**
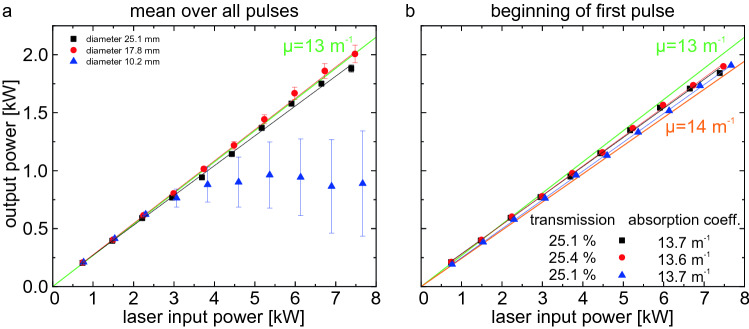
Output power as power in the bucket (aperture diameter 30 mm) as function of the input power. In (**a**), the mean value of the complete laser-on-time of three pulse trains (each 5 pulses of 0.1 s duration) is shown. Only for the larger spot diameters a linear behaviour is observed. In (**b**), only the power in the bucket at the beginning of the first pulse is plotted. Here, the power in the bucket is still a linear function with the input power for the smallest spot diameter. The green and orange lines are guide to the eyes for two different absorption coefficients.

In Fig. 5 the power in the bucket is plotted as function of the input power to evaluate the linearity of the transmission and to compare it with the literature values of the absorption coefficient. For the plot in (a), the mean values of the complete laser-on time of three pulse trains of five pulses each of 0.1 s duration are determined. As already discussed, the power in the bucket changes especially for the higher input powers and smaller spot diameters. The input power at which a deviation from linear behaviour begins is about 3 kW for the small spot size. The two larger diameters remain linear on average up to 7.5 kW input power. The deviation from the linear distribution is due to the distortion of the laser beam in the water and not to a non-linear absorption coefficient. If only the power in the bucket at the beginning of the first pulse is taken into account as done in Fig. 5b, the small spot diameter also shows a good linear behaviour up to 7.5 kW input power. Here the transmission is slightly above 25% for the water depth of 10.1 cm. This results in an absorption coefficient of $\mu = {13.7}\,{\mathrm{m}^{-1}}$, which is in good agreement with the literature^18, 29^.

For the larger spot diameters, the output power of the beginning of the first pulse is slightly smaller compared to the mean values over several pulses, showing that the power in the bucket increases within the pulses. This can be seen in Fig. 4a, where the power in the bucket for the 7.5 kW input power oscillates slightly in time. This slight change in transmission cannot be attributed to the temperature increase, as it was found that the absorbance marginally increases with increasing temperature^30^.

In conclusion of the experimental observation of the absorption in water it can be stated that the transmission is linear for the used parameters here with an absorption coefficient of $\mu = {13.7}\,{\mathrm{m}^{-1}}$. When analysing the temporal evolution, one observes increasing changes in the power in the bucket for higher laser powers and smaller spot sizes, hence for increasing intensities. When evaluating the power in the bucket as function of the input intensity (mean value within the 86%-criteria), one observes a threshold intensity of around 2.5 $\mathrm{kW/cm}^2$, where the transmission starts to decrease with increasing intensity for the complete pulse trains. If again only the values of the beginning of the first pulse are taken into account, no threshold is observed at least up to 8.0 $\mathrm{kW/cm}^2$.

#### Laser pulses of 5 s

**Figure 6:**
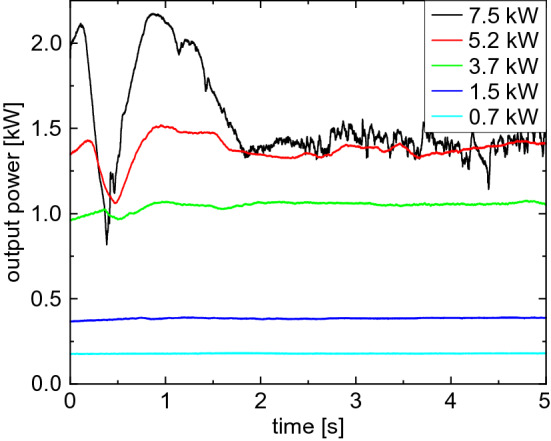
Temporal evolution of the output power as power in the bucket after propagation through 10.1 cm of water with $D4\sigma ={17.8}\,\mathrm {mm}$ and different input powers continuously for 5 s. For low input powers, a constant power in the bucket is observed, while for high input powers, a characteristic change with increasing magnitude is observed.

In comparison to the measurements of the transmitted intensity distribution and the refractive index changes, in Fig. 6 the temporal evolution of the power in the bucket for different input powers ($D4\sigma = {17.8}\,{mm}$) is shown for a continuous radiation for a duration of 5 s. For the lower two input powers, only minor changes in the power in the bucket are observed within the investigated time. Although a change in the transmitted intensity distribution has been observed already for 0.7 kW, the detected power in the bucket stays constant. The thermal blooming effect only redistributes the energy within the aperture of the detector. Hence, no significant thermal lensing effect occurs for low input powers.

For the higher input powers, on the other hand, significant changes do occur. For an input power of 7.5 kW, the power in the bucket within the first second first increases slightly before it shows a V-shaped valley. Subsequently, it decreases again until it stabilizes at a lower level than at the beginning. A similar behaviour is observed for 5.2 kW and 3.7 kW, however with a reduced amplitude and slower course. This behaviour can be explained with the thermal lensing as already discussed. With increasing input power, a stronger thermal lens arises, resulting in a stronger deflection of the laser beam from the primary path. For 7.5 kW, the Gaussian shape deforms to a ring within 0.1 s and then enlarges. After 0.2 s a reduced energy deposition (observed as small differential phase values because of comparably low water temperature, see details above) in the rear part of the water is observed. Hence, when the power in the bucket starts to decrease drastically, the main part of the laser beam gets deflected outside of the aperture of the detector by the thermal lens. At the minimum (at 0.5 s), the first macroscopic vapour bubbles also emerge (data not shown) resulting in additional scattering of the laser beam. After that, the water flow sets in, leading to a partial recovery of the power in the bucket, as at least the lower part of the laser path comes to a steady state. New cold water enters the laser path from the bottom getting heated up while flowing upwards. This also explains the bottom half moon shape of the transmitted intensity distribution.

The temporal evolution of the power in the bucket measured by the power meter (see Fig. 6) can be put into relation with the imaging of the intensity distribution (see Fig. 2). The linearity of the scattering intensity with power increase as well as the independence of a temperature increase of the scattering screen was confirmed in further experiments. To compare the scattering imaging with the power measurement, the scattering intensity was integrated on the complete screen and within an area representing the aperture of the power meter. For the lower two input powers (0.7 kW and 1.5 kW) again an almost constant integrated intensity is observed, irrespective of whether the complete scattering screen is taken into account or only a region corresponding to the aperture size of the power meter used. However, for the high input powers, a strong decrease is observed again after about 0.5s. This decrease is stronger if only the area of the aperture is considered compared to the entire screen.

## Conclusions

In this paper we showed that the absorption coefficient of tap water is $\mu ={13.7}\,{\mathrm{cm}^{-1}}$ and is constant for intensities at least up to 8.0 $\mathrm{kW/cm}^2$. However, the intensity distribution changes with time due to thermal blooming and thermal lensing even at low input powers. For low input powers, the transmitted intensity distribution change after propagation through 10.1 cm of water was similar to the well known change after propagating several kilometres in air. The thermal blooming emerges in a much shorter penetration depth due to the much higher absorption coefficient of water compared to air. For high input powers, however, first the establishment of a strong thermal lens was shown, because the onset of water movement is too slow. After the onset of a convective flow, a thermal blooming on a much higher level compared to the ones in air was shown. With the differential phase contrast of a Hartmann-mask multi-contrast imaging setup, changes of the refractive index distribution in the water phase were shown. This in-situ investigation of the water retrieved additional information emphasizing the origin of the thermal lensing and thermal blooming. The combination of the measurement of the transmission and the refractive index change allowed to confirm the validity of the corresponding simulations, with which further details of the water interior such as temperature distribution and medium movement velocity can be obtained, which are not accessible by experiments.
